# Impact of Infections in Patients Receiving Pembrolizumab-Based Therapies for Non-Small Cell Lung Cancer

**DOI:** 10.3390/cancers15010081

**Published:** 2022-12-23

**Authors:** Ethan A. Burns, Kelly Gee, Ryan B. Kieser, Jiaqiong Xu, Yuqi Zhang, Aubrey Crenshaw, Ibrahim N. Muhsen, Charisma Mylavarapu, Abdullah Esmail, Shivan Shah, Godsfavour Umoru, Kai Sun, Carlo Guerrero, Zimu Gong, Kirk Heyne, Monisha Singh, Jun Zhang, Eric H. Bernicker, Maen Abdelrahim

**Affiliations:** 1 Houston Methodist Neal Cancer Center, Houston Methodist Hospital, 6445 Main St. Outpatient Center, Floor 24, Houston, TX 77030, USA; 2 Department of Medicine, Houston Methodist Hospital, 6565 Fannin St., Smith Tower, Floor 10, Houston, TX 77030, USA; 3 Center for Health Data Science and Analytics, Houston Methodist Research Institute, 7550 Greenbriar RB4-129, Houston, TX 77030, USA; 4 Section of Hematology and Oncology, Department of Medicine, Baylor College of Medicine, 7200 Cambridge St. 7th Fl, Houston, TX 77030, USA; 5 Section of Hematology and Oncology, Scripps Health, 10666 N. Torrey Pines Rd., La Jolla, CA 92037, USA; 6 Department of Infectious Diseases, Houston Methodist Hospital, 6550 Fannin St., Ste 1101, Houston, TX 77030, USA; 7 Department of Pharmacy, Houston Methodist Hospital, 6565 Fannin St, Houston, TX 77030, USA

**Keywords:** infection, pembrolizumab, non-small cell lung cancer, rate, outcomes

## Abstract

**Simple Summary:**

Infections are reported in up to 19% of pembrolizumab treated patients across clinical trials and in 7–100% in the standard of care population receiving various immune checkpoint inhibitor therapies. Risk factors for infection development remains controversial but among them include immunosuppression for the management of immune related adverse events. This study assessed the incidence, type, and impact of infection on morbidity and mortality in non-small cell lung cancer patients receiving pembrolizumab-based therapies. Infections occurred in 46% patients, resulted in a 70% hospitalization rate, treatment delays in 50%, and poorer median overall survival. Furthermore, poorer performance status and anti-infective use at therapy initiation were associated with a higher risk of developing an infection. Infections should be uniformly reported across clinical trials. Anti-infective stewardship may be beneficial to augment treatment adherence and efficacy.

**Abstract:**

Background: Immune checkpoint inhibitor (ICI) therapy has significantly improved outcomes across a range of malignancies. While infections are a well-known contributor to morbidity and mortality amongst patients receiving systemic chemotherapy regimens, little is known about the impact of infections on patients receiving ICI therapy. This study aims to assess incidence, risk factors, and outcomes in patients who develop infections while on pembrolizumab-based therapies for non-small cell lung cancer (NSCLC). Methods: Patients receiving pembrolizumab for stage III/IV NSCLC from 1/1/2017-8/1/2021 across seven hospitals were identified. Incidence and type of infection were characterized. Covariates including baseline demographics, treatment information, treatment toxicities, and immunosuppressive use were collected and compared between infected and non-infected patients. Outcomes included the rate of infections, all-cause hospital admissions, median number of treatment cycles, overall survival (OS), and progression free survival (PFS). Univariable and multivariable analysis with reported odds ratio (OR) and 95% confidence intervals (CI) were utilized to evaluate infection risks. OS and PFS were analyzed by Kaplan–Meier analysis and tested by log-rank test. *p*-value < 0.05 was considered statistically significant. Results: There were 243 NSCLC patients that met the inclusion criteria. Of these, 111 (45.7%) had one documented infection, and 36 (14.8%) had two or more. Compared to non-infected patients, infected patients had significantly more all-cause Emergency Department (ED) [37 (33.3%) vs. 26 (19.7%), *p* = 0.016], hospital [87 (78.4%) vs. 53 (40.1%), *p* < 0.001], and ICU visits [26 (23.4%) vs. 5 (3.8%), *p* < 0.001], and had poorer median OS (11.53 [95% CI 6.4–16.7] vs. 21.03 [95% CI: 14.7–24.2] months, *p* = 0.033). On multivariable analysis, anti-infective therapy (OR 3.32, [95% CI: 1.26–8.76], *p* = 0.015) and ECOG of >1 (OR 5.79, [95% CI 1.72–19.47], *p* = 0.005) at ICI initiation conferred an increased risk for infections. At last evaluation, 74 (66.7%) infected and 70 (53.0%) non-infected patients died (*p* = 0.041). Conclusion: Infections occurred in nearly half of patients receiving pembrolizumab-based therapies for NSCLC. Infected patients had frequent hospitalizations, treatment delays, and poorer survival. ECOG status and anti-infective use at ICI initiation conferred a higher infection risk. Infection prevention and control strategies are needed to ameliorate the risk for infections in patients receiving ICIs.

## 1. Background

Immune checkpoint inhibitor (ICI) therapy has changed the therapeutic framework and improved outcomes across a range of malignancies in the neoadjuvant, adjuvant, and metastatic settings. In non-squamous NSCLC, long term follow-up of the pivotal Keynote 021 study found that the combination of carboplatin and pemetrexed with pembrolizumab yielded superior OS and PFS versus chemotherapy alone, regardless of programmed death ligand-1 (PDL-1) expression [1]. In the Keynote-407 study assessing outcomes of carboplatin and either paclitaxel or nanoparticle albumin-bound [nab]-paclitaxel with or without pembrolizumab in squamous NSCLC, the patients randomized to the pembrolizumab arm had significantly longer OS and PFS compared to patients receiving chemotherapy alone [2]. Furthermore, pembrolizumab has also been established and approved as a monotherapy following the results reported in the Keynote-024 study which demonstrated superior OS and PFS compared to platinum-based chemotherapy in the cohort with a PDL-1 status ≥50% and has also shown beneficial outcomes as monotherapy with TPS > 1% in the elderly population [3,4].

Immune related adverse events (irAE) are a well-known complication of therapy and occur in up to 25–30% of NSCLC patients [1,2,3,4]. IrAE can impact virtually any organ and are fatal in 0.8–1.3% of patients [5]. While these toxicities are well described, the full gambit of toxicities in this growing treatment population needs to be further explored to maximize therapeutic adherence. Infections, a common cause of morbidity and mortality in patients with solid organ malignancies, are infrequently reported in clinical trials of ICI therapy. Furthermore, while several recent retrospective studies have assessed the incidence of infections in ICI-treated patients, the rate varied dramatically, ranging from 7.4–100% [6,7,8,9,10]. While these studies do not imply causality, they suggest that infections may not be infrequent while on ICI therapy. However, questions remain, including the rate and burden of infections with specific ICIs and malignancies, as well as the effect these infections have on treatment adherence, morbidity and mortality. The aim of this study is to assess the rate and outcomes of infections that occur in NSCLC patients receiving pembrolizumab-based therapies.

## 2. Materials/Methods

### 2.1. Study Design

This is an institutional review board (IRB) approved, observational, retrospective study assessing the rate, risk factors, and outcomes of infections in patients receiving pembrolizumab-based systemic therapy regimens for the treatment of advanced or metastatic NSCLC. Electronic charts of individuals who received pembrolizumab between the dates of 1 January 2017–1 August 2021 across 7 academic and community hospitals in a single hospital network were reviewed. Patients were included if they were ≥18 years of age at the time of diagnosis, pathologic diagnosis was available, had a confirmed receipt of ≥1 cycle of pembrolizumab either as monotherapy or in combination with a systemic chemotherapy regimen at a study institution, and if patients had stage III or IV disease at the time of ICI initiation. Patients were excluded if they had missing information including confirmed diagnosis, available treatment records, therapy at an institution outside of the hospitals approved for this study, or stage I/II disease.

### 2.2. Data Collection and Outcomes

All patient data was individually reviewed for study inclusion. A patient was considered to have an infection if there was relevant microbiological confirmation, or if the patient had clinical findings including symptoms correlated with laboratory evidence or imaging characteristics suspicious for infection. Patients with ambiguous infection status were independently reviewed by an infectious disease specialist. If the patient developed relevant symptoms indicative of infection documented in the outpatient setting, they were included. If no infection was documented anywhere in chart review, then they were considered to not have an infection.

Patients were grouped into the infection cohort if they had an infection while on active treatment with pembrolizumab or within 3 months following discontinuation of ICI therapy and not actively receiving other therapy at the time the infection was diagnosed. Infections were divided into bacterial, viral, fungal, parasitic, and systematically categorized into intracranial, intraabdominal, pulmonary, skin and soft tissue infection (SSTI), urinary tract infection (UTI), head and neck, bacteremia, cardiac, neutropenic fever, COVID-19, and presumed sepsis. If a specific pathogen was isolated, it was recorded and catalogued based on infectious site. The course of each infection, including whether the infection resulted or contributed to an emergency department (ED), inpatient, or intermediate unit (IMU)/intensive care unit (ICU) stay was reported. In addition, the impact on treatment delays, interruptions, discontinuation, or death were recorded. If an infection was suspected to be the direct cause of death, it was assessed by an infectious disease specialist for corroboration. Any uncertainties in treatment management were reviewed by a licensed pharmacist in the Cancer Center.

Additional covariates were collected and compared between infected and noninfected patients, which included age, sex (male/female), baseline medical comorbidities, Eastern Cooperative Oncology Group (ECOG) performance status (ranging from 0 defined as fully active without restrictions to 4, which is completely disabled), race (self-reported), chronic infections (including history of Hepatitis B virus [HBV], Hepatitis C virus [HCV], Human Immunodeficiency Virus [HIV], genital or cutaneous herpes simplex virus infections, tuberculosis, cytomegalovirus, and varicella zoster reactivation), line of therapy in which pembrolizumab was administered (1L, 2L, >2L), monotherapy/combination therapy, anti-infective use at ICI initiation, and growth factor use.

The primary objective of this study was to determine the rate of infections in patients receiving ICI therapy for NSCLC. Secondary objectives included: categorical type of infection; the impact of infections on cancer-related treatment and outcomes; impact on morbidity (defined as all-cause emergency department, inpatient hospitalizations, or IMU/ICU visits); infection-related death; impact on overall survival (OS)/progression free survival (PFS).

## 3. Statistical Analysis and Outcomes

Baseline characteristics were reported as means ± SD for continuous variables and frequencies and percentages for categorical variables by infection status (infected vs. non-infected). Time to infection was reported as median (range: minimum to maximum) in days. Continuous variables were compared by Student’s *t*-test or Mann–Whitney test, and categorical variables were compared by Chi-square test or Fisher’s exact test. Univariable and multivariable logistic regression models were used to establish characteristics that were independently associated with infectious risk during ICI therapy. These outcomes were reported as odds ratio (OR), along with 95% confidence intervals (CI) and *p*-values. Kaplan–Meier analysis was performed between infected or non-infected patients, and log-rank test was used to test the OS and PFS between the two cohorts. Cox proportional hazard ratios reported with associated hazard ratio (HR) and 95% CI were used to assess factors impacting OS and PFS with univariable and multivariable models. All analyses were performed with STATA version 17 (StataCorp. 2021. Stata Statistical Software: Release 17. StataCorp LLC, College Station, TX, USA). Statistical significance was defined as two-tailed *p* < 0.05 for all tests.

## 4. Results

### 4.1. Baseline Factors

**Figure 1:**
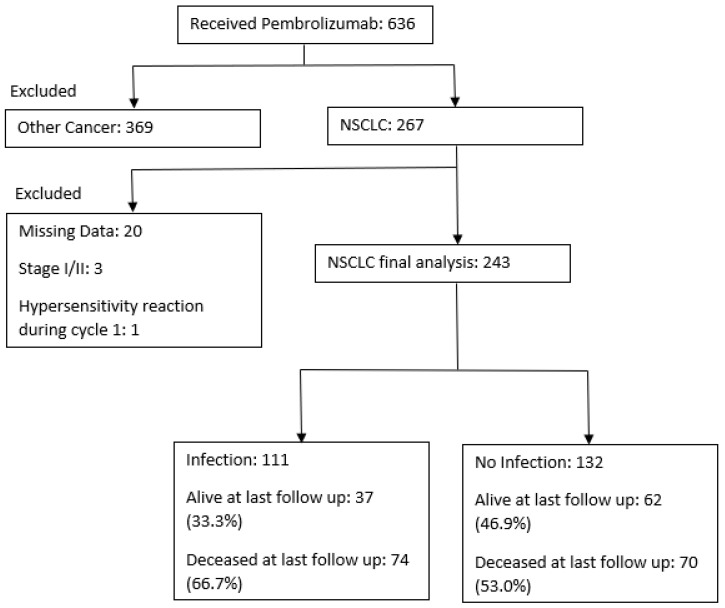
Consort diagram. NSCLC: Nonsmall cell lung cancer. Missing data includes patients who may have received treatment at one of the seven hospitals but did not have electronic charting information available including progress notes, labs, or treatment notes. The one patient with a hypersensitivity reaction during cycle 1 did not complete a full infusion, and was not included.

Between 1 January 2017 and 1 August 2021, there were 810 evaluable patients, of which 243 patients had NSCLC and met inclusion criteria for analysis (Figure 1). Of these, 111 (45.7%) had at least one infection. Among those infected, there were 93 (83.8%) with adenocarcinoma, 15 (13.5%) with squamous cell carcinoma, and 3 (2.7%) with “other” histology. The mean age of infected patients was 69.12 ± 11 years, 59 (53.15%) were female, 83 (74.77%) identified themselves as white, and the most common pre-existing medical conditions included chronic obstructive pulmonary disease (COPD) in 49 (44.1%) and type 2 diabetes mellitus (T2DM) in 26 (23.42%). Most infected patients had an ECOG of 1 or 2 in 47 (42.34%) and 23 (20.72%), respectively. Out of the 243 patients, 22 (9.0%) had one or more chronic infections at ICI initiation, 15 (13.5%) of which went on to have an infection. Of these 15, 2 (13.3%) had HBV, 4 (26.7%) had HCV, 1 (6.7%) had HIV, 3 (20.0%) had a history of tuberculosis, 7 (46.7%) had HSV, and 1 (6.7%) had HIV. Baseline PD-L1 activity was evaluable in 95 (85.6%) patients with infection; 39 (35.14%) had absent membranous staining, 19 (17.1%) had staining ranging from 1–20%, and 37 (33.33%) had >20% PD-L1 activity.

There were 41 (36.94%) infected patients compared to 39 (29.55%) noninfected on monotherapy, and 70 (63.06%) and 93 (70.45%) on multiagent systemic therapy, respecively. Over 70% of patients in both cohorts were using pembrolizumab-based therapies in 1L. A total of 27 (24.3%) and 38 (28.8%) in infected and non-infected cohorts, respectively required radiation while on an ICI over the course of their disease. At initiation of ICI therapy, 31 (12.7%) patients were on steroids, and 15 (48.4%) of these patients developed infections. Moreover, 21 (18.92%) infected patients were on anti-infectives at the time of ICI initiation (Table 1).

### 4.2. Infections

**Figure 2:**
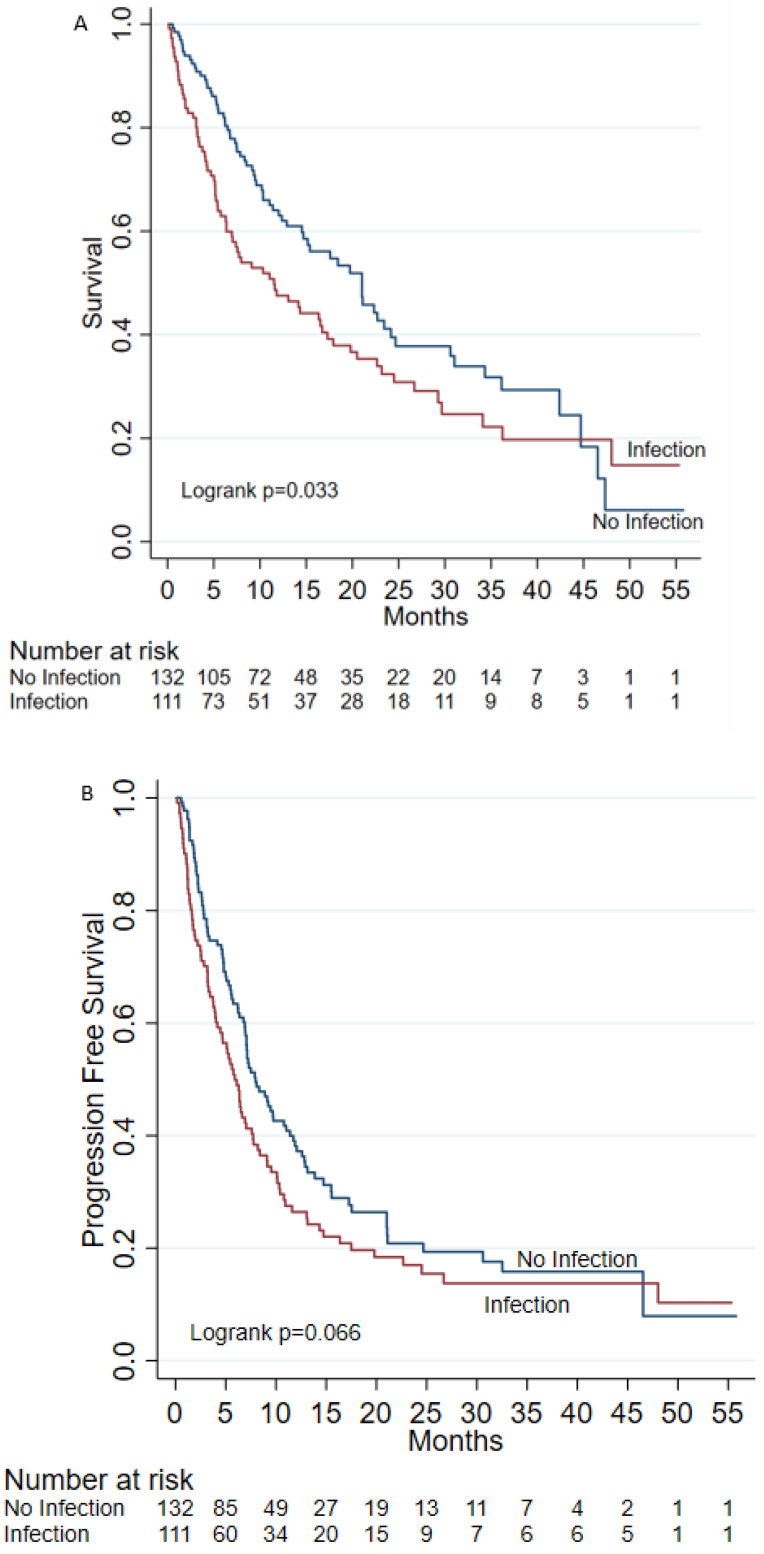
(**A**) Median overall survival. (**B**) Progression free survival.

Of the 243 patients included, 111 (45.7%) had one infection, 55 (22.6%) had two infections, and 18 (7.4%) had three or more infections (Table 2, Figure 2 and Figure S1). Median time to first, second, and third infection was 58 (1–1465), 124 (6–844), and 213.5 (25–1414) days from initiation of therapy, respectively. IMU/ICU admission as a result of complications arising from infection was required in 13 (11.7%) with one infection, 14 (25.4%) with 2 infections, and 6 (33.3%) patients with ≥3 infections. An infectious organism was confirmed in 53 (47.7%) with one infection, 22 (40.0%) with two infections, and 6 (33.3%) with ≥3 infections; a summary of these organisms can be found in Table S1 of the Supplementary Appendix. Of note, *Escherichia coli* spp. and *Clostridioides difficile* were the most commonly isolated bacteria occurring in 11 (9.9%) and 5 (4.5%) patients, while *Rhinovirus/Enterovirus*, *varicella zoster* reactivation, and *Herpes simplex* 1/2 orogenital infections were the most commonly isolated viral infections, occurring in 4 (3.6%), 3 (2.7%), and 3 (2.7%) patients, respectively. The most common sites of infection included pulmonary in 48 (43.2%), urinary tract in 20 (18.0%), and SSTI in 15 (13.5%) patients (Figure S1). COVID-19 pneumonia was diagnosed in 3 (2.7%) patients while receiving pembrolizumab. There were a total of 7 (6.3%) fungal infections isolated, of which 3 (42.8%) were on monotherapy and 4 (57.1%) were on combination therapy. Of the 15 patients with chronic infections, 3 patients with known HSV developed reactivation with orogenital infections; no other reactivation of infections occurred, including in those not on active therapy for their disease at the time (Table S1).

For treatment, 104 patients (93.7%) received antimicrobials, 12 (10.8%) received antiviral therapy, and 10 (9.0%) received antifungal therapy. The most commonly prescribed antibiotic classes were cephalosporins and vancomycin, prescribed 64 (29.6%) and 53 (24.5%) times each, respectively (Table S2). A total of 47 (42.3%) patients had an infectious disease consultation at least once.

On univariable analysis, COPD (*p* = 0.013), and ECOG performanc status of 1 (*p* = 0.027), 2 (*p* = 0.001), or unknown (*p* = 0.001) ECOG status, history of chronic infection (*p* = 0.032), and anti-infective at time of ICI initiation (*p* = 0.003) were associated with higher risk of developing infection. However, on multivariable analysis, only ECOG 2 (OR 5.79 [95% CI 1.72, 19.47], *p* = 0.005), unknown ECOG (4.80 [95% CI 1.57, 14.74], *p* = 0.006), and anti-infective use at ICI initiation (OR 3.32 [95% CI 1.26, 8.76], *p* = 0.015) retained a statistically significant risk of developing an infection while on ICI therapy (Table 3). 

### 4.3. Outcomes

A total of 66 (27.16%) patients developed an irAE event, of which 33 (29.73%) occurred in the infection cohort (*p* = 0.41) (Table 4). Pneumonitis was more common in the infected cohort on univariable analysis (*p* = 0.009). Over the course of workup or management of documented infections, treatment delays occurred in 56 (50.4%), treatment discontinuation in 28 (25.2%), and disease progression was documented in 16 (14.4%) patients (Table S2, Figure S1). There were significantly more all-cause ED [37 (33.3%) vs. 26 (19.7%), *p* = 0.016], hospital [87 (78.38%) vs. 53 (40.15%), *p* < 0.001], and IMU/ICU visits [26 (23.42%) vs. 5 (3.79%), *p* < 0.001] in infected compared to non-infected patients, respectively. At the last follow up, there were a total of 144 (59.26%) deaths, of which 74 (66.7%) and 70 (53.03%) occurred in infected and noninfected, respectively (*p* = 0.031). Of these, 11 (14.86%) with infections died due to complications arising from the infection; 4 (5.71%) in the noninfected group died due to eventual infection but were not included in the infection cohort because they had an infection while on subsequent treatment or had an infection >3 months after ICI discontinuation (Table 4).

Regarding timing of infections, 30/111 (27.0%) developed an infection during the first two cycles of therapy, which comprised 54.5% of the 55 patients who received only two cycles of therapy. Moreover, 6/11 (54.5%) of the patients that died as a direct cause of infection died after receiving one (*n* = 5) or two (*n* = 1) cycles. There were 11 patients with infections that had disease progression within the first two cycles, of which seven (63.6%) died from primary disease and one (9.1%) died from infection. There were another 23 that received the first two cycles who did not have disease progression reported; however 10 (43.5%) deaths were due underlying primary disease burden, five (21.7%) were due to infection, and two (8.7%) had multiorgan failure.

Median OS was 11.53 [95% CI 6.37–16.73] months for infected and 21.03 [95% CI 14.7–24.17] months for non-infected (*p* = 0.033) (Figure 2A). Median OS for patients on monotherapy and no infection was 22.33 (95% CI 11.4–46.53) months compared to monotherapy with infection which was 14.17 months (95% CI: 5.4–24.5), and for patients on multiagent therapy, was 19.73 months (95% CI: 12.03–31.03) without an infection, and 10.33 months (95% CI 5.77–16.53) with an infection (Figure S2). However, when controlling for treatment and infection status, these differences were not statistically significant (*p* = 0.12). Of note, while 47 (42.3%) of infected patients required an infectious disease consultation, this did not significantly impact survival (Figure S2B). Median PFS in infected and non-infected cohorts was 5.9 (95% CI 4.16, 7.6) and 7.9 (95% CI 6.94, 11.03) (*p* = 0.066) months, respectively (Figure 2B). On multivariable analysis, age (HR 1.02 [95% CI 1.00, 1.04], *p* = 0.019), T2DM (HR 1.58 [95% CI 1.07, 2.33], *p* = 0.022), and ECOG 2 (HR 2.34 [95% CI 1.08, 5.08], *p* = 0.031), had a higher mortality rate, whereas patients that received 3 or more cycles of treatment (HR 0.33 [95% CI 0.20, 0.53], *p* < 0.001) had better OS. With regard to PFS, COPD (HR 0.59 [95% CI 0.40, 0.88], *p* = 0.010) had a better reported PFS, whereas patients on 2L (HR 1.74 [95% CI 1.15, 2.63], *p* = 0.009) and >2L (HR 2.28 [95% CI 1.20, 4.33], *p* = 0.011) had higher rate of progression compared to patients receiving pembrolizumab in 1L.

## 5. Discussion

Infections impart a substantial risk for morbidity and mortality in patients with solid organ malignancies. However, there is scarce data assessing the burden and impact of infections occurring with specific ICI therapies. We recently reported upon outcomes in patients receiving pembrolizumab for head and neck squamous cell carcinomas (HNSCC) and urothelial adenocarcinomas [11,12], indicating that infections increase the number of all-cause hospitalizations and are associated with poorer survival. Herein, we report the rate and impact of infections in patients with NSCLC who received pembrolizumab. The following key findings were found: infections occurred in nearly half of patients, with the most common subtype of infection being bacterial pulmonary infections; anti-infective use and poorer ECOG performance status at pembrolizumab initiation were associated with a higher risk of infection; infected patients had significantly higher rate of all-cause ED, hospital, and IMU/ICU admissions; infection status negatively impacted OS and resulted in death in approximately 10% of patients.

The risk and impact of infection in patients receiving ICI therapy remains controversial. A recent review of trial data by the European Society of Clinical Microbiology and Infectious Diseases (ESCMID) led to the conclusion that that CTLA-4, PD-1, and PD-L1 inhibitors are not independently associated with risk of infection [13]. However, the package insert for pembrolizumab cautions that genitourinary tract infections are reported in up to 19% of patients, upper respiratory tract in 13–44%, and pneumonia in 12–19% of patients [14]. While clinical trials are the gold standard for determining treatment efficacy, they may not adequately assess infrequent toxicities, particularly if causality between the medication and event is not clearly established. Post-marketing studies are helpful in these cases to assess outcomes of less common toxicities in the standard of care population that may be more heterogenous than the trial cohorts. Until recently, few studies have assessed the spectrum, burden, and impact of infections. In 2016, an analysis of infection occurrence in melanoma patients receiving nivolumab or pembrolizumab with or without ipilimumab determined that the rate of “serious” infections requiring hospitalization occurred in 7.3% of cases [8]. In a recent meta-analysis that assessed over 21,000 patients from over 36 studies, ICI monotherapy had similar risks for all-grade infections (relative risk [RR] = 1.02; 95% CI 0.84–1.24; *p* = 0.85), a lower risk of infection compared to chemotherapy alone (RR = 0.58, 95% CI 0.4–0.85; *p* = 0.01), and a higher risk than chemotherapy alone when combined with chemotherapy (RR = 1.37, 95% CI 1.23–1.53; *p* < 0.01) or when dual-ICI therapy was used 2.26 (95% CI 1.34–3.8; *p* < 0.01) [9]. In a study of 111 patients assessing infections in different ICI therapies for a range of cancers, 24% had a suspected or confirmed bacterial infection, but only one viral infection was reported [7]. In a French chart review, the infection rate was 18%, with most common infections being pneumonia in 58%, SSTI and UTI occurring in 19.4% each [10]. In our study, 46% of NSCLC receiving pembrolizumab developed an infection, resulting in a hospitalization rate of nearly 70%. This suggests a highly heterogenous population across post-marketing studies, likely stemming from age, ECOG, baseline demographic factors, and infection severity. This analysis only assessed the NSCLC population treated with pembrolizumab-based therapies, but our recent reports assessing HNSCC and urothelial adenocarcinomas reported similar rates of infection and hospitalization rates [11,12]. Similar to other studies, pulmonary infections, SSTI, and UTIs were the most common infections, but our analysis found a higher number of viral and fungal infections.

This study was not designed to assess causality between pembrolizumab and infections, but rather to assess the rate and risk of infections in NSCLC patients receiving pembrolizumab-based therapies. Risk factors for developing infection in this study included a poorer ECOG performance status and anti-infective use at pembrolizumab initiation. Similar findings were also seen in patients receiving pembrolizumab for HNSCC [12]. Notably, our study did not find an increased risk for infection in patients receiving concomitant chemotherapy, radiation therapy, or in patients who had immunosuppressive therapy at the initiation of pembrolizumab or who received immunosuppression to manage irAE. Patients with irAE may be at increased risk for infection by several mechanisms, including immune dysregulation stemming from ICI therapy, local tissue damage, or from immunosuppressive therapies used to ameliorate effects of irAE, such as corticosteroids, infliximab, cyclophosphamide, and mycophenolate mofetil [15]. Sutthichai et al. evaluated the incidence of infection in those on ICI therapy in solid tumor and hematologic malignancies and found that 68% of patients were found to have de novo infections while on an ICI, and all of these patients were started on immunosuppression for irAEs. The study suggested that some patients on ICI therapies, whether as a consequence of the ICI or specifically irAEs, will develop infections [6]. Several other recent studies found that immunosuppressive use, particularly systemic corticosteroids, predisposed patients to infections [7,8,9]. Conversely, like the present study, there are other findings that would suggest factors aside from immunosuppressive use may drive the risk of infection development [10,11]. It has been proposed that immunity dysregulation can occur with the excessive immune response from ICIs and that this in of itself can favor development of infections [9]. Moreover, polymorphisms in PD-1, PD-L1, and CTLA-4 have been associated with the development of a diverse array of infections, suggesting a direct correlation between immune dysregulation and infection development [9,16,17,18].

Our study found that anti-infective use at ICI initiation was associated with a higher infection risk. There is a growing body of literature that anti-infective use negatively impacts ICI efficacy. It is feasible that the association between the higher infection rate in our study stems from an iatrogenic alteration of the normal intestinal microbiota. The intestinal microbiome has several vital functions including the maturation of host immune responses, antigenic recognition, providing protection against bacterial overgrowth, modifying specific drugs, and removing toxins [19]. While individual responses to ICI therapy is derived from a combination of immune competency, diversity, and adequate antigenic variation, recognition, and expression, the intestinal microbiota has recently been found to also play a pivotal role, which arises from the development of tumor antigenicity, adjuvanticity, and bystander activation through an enhanced inflammatory state in the tumor micro-environment [19,20,21,22]. Anti-infectives produce gut micriobiota dysbiosis resulting in loss of diversity, alteration in the basal metabolic capactity, and possible introduction of invasive pathogens [23]. While these changes occur within several days of antimicrobial exposure, it may take up to 6 weeks or more for the intestinal flora to recover [24]. These dramatic changes also diminish the response of cytotoxic T-cells to cancer antigens, and data suggests that these patients have inferior responses to anti-PD-1 ICI therapy and have a lower taxonomic intestinal diversity [25,26,27]. A recent meta-analysis demonstrated poorer OS in patients that received antimicrobials in the month preceding (hazard ratio [HR] 1.64, 95% CI 1.27–2.11) ICI or in the month following ICI therapy (HR 2.36, 95% CI 1.66–2.33), as well as poorer PFS (HR 1.52, 95% CI 1.36–1.70), with the latter being more prominent in patients with RCC or melanoma than with NSCLC [20]. Multiple other studies assessing outcomes of antibiotics in NSCLC treated with ICI therapies have shown negative impacts on OS and PFS, primarily in patients receiving an anti-infective in the preceding 6 weeks up to the first 4 weeks of anti-PD-1 ICI initiation [28,29,30,31,32,33,34]. It is possible that the poorer outcomes in the infected group of this study was similarly impacted. However, while PFS was numerically lower in our cohort, it was not significantly different than the non-infection cohort. This may be explained by the higher early mortality rate stemming from multiorgan failure and infections in the infection cohort. With this accumulating data, anti-infective stewardship, a frequently underutilized strategy in healthcare, may be beneficial to minimize unnecessary anti-infective use. Replenishment of the intestinal microbiome may also be a feasible strategy, particularly in the infected population. A recent meta-analysis exploring the clinical impact of probiotics in patients with NSCLC who received ICIs reported a superior OS (HR = 0.50, 95% CI: 0.30–0.85, *p* = 0.01) and PFS (HR = 0.51, 95% CI: 0.42–0.61, *p* < 0.01), suggesting this may be a viable strategy [35].

Limitations of this study should be recognized. As a retrospective chart review, there will be biases intrinsic to the study design itself. Similar to the limited number of other studies currently available, our reported number of infections were notably higher indicating heterogeneity across study populations, likely stemming from underlying population-level differences in baseline comorbidities, performance status, and treatment. In addition, several analyses were based on small sample sizes and as such this led to wide CIs and may have impacted the true clinical significance of the outcomes reported. Furthermore, we did not have enough information or a population large enough to perform a subgroup analysis on the class of anti-infective used, duration of utilization, route of administration, or type of chronic infection. Finally, given the retrospective nature of this chart review study, patients were excluded from the analysis if they did not have relevant information including progress notes, imaging studies, or therapy records (*n* = 20). This exclusion may have impacted the final results of the multivariable analysis as well as survival differences given the current sample size.

## 6. Conclusions

Infections in patients receiving pembrolizumab-based therapies for NSCLC are common and result in significantly higher rates of all-cause ED, hospital, and IMU/ICU admissions. Furthermore, infections resulted in death in 10% of infected patients and shorter median OS compared to non-infected patients. Risks for infection development included poorer ECOG performance status and anti-infective use at initiation of pembrolizumab. Studies to assess the benefit of anti-infective stewardships and reduction of anti-infective use at initiation of ICI therapy are needed.

## Figures and Tables

**Table 1:** Baseline demographics.

	Infection			
	Yes	No	Total	*p*-Value
	*N* = 111	*N* = 132	*N* = 243	
Age	69.12 ± 11.00	67.68 ± 11.28	68.34 ± 11.15	0.32
Female	59 (53.15%)	56 (42.42%)	115 (47.33%)	0.095
Race				0.85
White	83 (74.77%)	97 (73.48%)	180 (74.07%)	
Black	18 (16.22%)	20 (15.15%)	38 (15.64%)	
Other Comorbidities	10 (9.01%)	15 (11.36%)	25 (10.29%)	
T2DM	26 (23.42%)	34 (25.76%)	60 (24.69%)	0.67
CAD	33 (29.73%)	27 (20.45%)	60 (24.69%)	0.095
ESRD	1 (0.90%)	0 (0.00%)	1 (0.41%)	0.27
COPD	49 (44.14%)	38 (28.79%)	87 (35.80%)	0.013
IV Drug Use	0 (0.00%)	1 (0.76%)	1 (0.41%)	0.36
Alcoholism	5 (4.50%)	10 (7.58%)	15 (6.17%)	0.32
Chronic Indwelling Catheter	3 (2.70%)	1 (0.76%)	4 (1.65%)	0.24
Smoking Status				0.30
Never	13 (11.71%)	21 (15.91%)	34 (13.99%)	
Previous	81 (72.97%)	84 (63.64%)	165 (67.90%)	
Current	17 (15.32%)	27 (20.45%)	44 (18.11%)	
ECOG				0.001
0	5 (4.50%)	23 (17.42%)	28 (11.52%)	
1	47 (42.34%)	67 (50.76%)	114 (46.91%)	
2	23 (20.72%)	13 (9.85%)	36 (14.81%)	
3	3 (2.70%)	3 (2.27%)	6 (2.47%)	
4	0 (0.00%)	1 (0.76%)	1 (0.41%)	
Unknown	33 (29.73%)	25 (18.94%)	58 (23.87%)	
Previous ICI therapy	6 (5.41%)	8 (6.06%)	14 (5.76%)	0.83
History of Chronic Infections	15 (13.51%)	7 (5.30%)	22 (9.05%)	0.026
NSCLC Subtype				
Adenocarcinoma	93 (83.8%)	101 (76.5%)	194 (79.8%)	0.366
Squamous Cell	15 (13.5%)	24 (18.2%)	39 (16.0%)	
Other	3 (2.7%)	7 (5.3%)	10 (4.1%)	
PD-L1 Activity				0.78
Absent	39 (35.14%)	48 (36.36%)	87 (35.80%)	
1–10%	13 (11.71%)	22 (16.67%)	35 (14.40%)	
11–20%	6 (5.41%)	5 (3.79%)	11 (4.53%)	
>20%	37 (33.33%)	38 (28.79%)	75 (30.86%)	
Unknown	16 (14.41%)	19 (14.39%)	35 (14.40%)	
Therapy				0.27
Monotherapy	41 (36.94%)	39 (29.55%)	80 (32.92%)	
Combination with Chemotherapy	70 (63.06%)	93 (70.45%)	163 (67.08%)	
Radiation Therapy	27 (24.3%)	38 (28.8%)	65 (26.7%)	0.469
Surgery				0.097
No Surgery	90 (81.08%)	95 (71.97%)	185 (76.13%)	
Primary Surgery	9 (8.11%)	23 (17.42%)	32 (13.17%)	
Secondary Surgery	12 (10.81%)	14 (10.61%)	26 (10.70%)	
Number of Cycles				
Total (median)	5.00 (2.00-13.00)	8.00(4.00–12.00)	7.00 (3.00–12.00)	0.057
Distribution of cycles				0.067
1	20 (18.02%)	11 (8.33%)	31 (12.76%)	
2	10 (9.01%)	11 (8.33%)	21 (8.64%)	
≥3	81 (72.97%)	110 (83.33%)	191 (78.60%)	
Line of Therapy				0.60
1L	85 (76.58%)	93 (70.45%)	178 (73.25%)	
2L	21 (18.92%)	31 (23.48%)	52 (21.40%)	
>2L	5 (4.50%)	8 (6.06%)	13 (5.35%)	
Antibiotics at time of ICI initiation	21 (18.92%)	8 (6.06%)	29 (11.93%)	0.002
GCSF Use	12 (10.81%)	16 (12.12%)	28 (11.52%)	0.75

ICI: Immune checkpoint inhibitor. T2DM: Type 2 diabestes mellitus; CAD: Coronary artery disease; COPD: Chronic obstructive pulmonary disease; ECOG: Eastern Cooperative Oncology Group; GCSF: Granulocyte colony stimulating factor. 1L: First line; 2L: second line.

**Table 2:** Distribution, frequency, timing of infections.

	1st Infection	2nd Infection	3rd Infection
Total	111	55	18
Bacteremia	8 (7.2%)	3 (5.4%)	0 (0.0%)
COVID-19	3 (2.7%)	0 (0.0%)	0 (0.0%)
Head and Neck	5 (4.5%)	3 (5.4%)	0 (0.0%)
Intra-abdominal	6 (5.4%)	3 (5.4%)	1 (5.6%)
Intracranial	0 (0.0%)	1 (1.8%)	0 (0.0%)
Neutropenic Fever	3 (2.7%)	0 (0.0%)	2 (11.1%)
Presumed Sepsis	3 (2.7%)	3 (5.4%)	1 (5.6%)
Pulmonary	48 (43.2%)	28 (50.9%)	9 (50.0%)
SSTI	15 (13.5%)	5 (9.1%)	4 (22.2%)
Urinary	20 (18.0%)	9 (16.4%)	1 (5.6%)
Type of Pathogen			
Bacterial	90 (81.1%)	50 (90.9%)	15 (83.3%)
Virus	18 (16.2%)	2 (3.6%)	2 (11.1%)
Fungal	3 (2.7%)	3 (5.4%)	1 (5.6%)
Culture Data Available	51 (45.9%)	21 (38.2%)	6 (33.3%)
Result of Infection			
ED Visits	6 (5.4%)	3 (5.4%)	0 (0.0%)
Inpatient Admission	78 (70.3%)	36 (65.4%)	11 (61.1%)
IMU/ICU Admission	13 (11.7%)	14 (25.4%)	6 (33.3%)
Outpatient	14 (12.6%)	3 (5.4%)	1 (5.6%)
Median time to infection (days)	58 (0–1465)	124 (6–844)	213.5 (25–1414)

SSTI: Skin and soft tissue infection.

**Table 3:** Univariable and Multivariable Analysis.

	Univariable		Multivariable	
	OR (95% CI)	*p*-Value	OR (95% CI)	*p*-Value
Age	1.01 (0.99, 1.04)	0.319		
Female	1.54 (0.93, 2.56)	0.096	1.52 (0.86, 2.70)	0.153
T2DM	0.88 (0.49, 1.59)	0.674		
CAD	1.65 (0.91, 2.96)	0.096	1.53 (0.79, 2.95)	0.204
COPD	1.96 (1.15, 3.33)	0.013	1.59 (0.88, 2.85)	0.121
Alcoholism	0.58 (0.19, 1.74)	0.327		
Chronic indwelling catheter1y	3.64 (0.37, 35.49)	0.266		
Smoking Status				
Never	Reference			
Previous	1.56 (0.73,3.32)	0.251		
Current	1.02 (0.41,2.55)	0.971		
ECOG				
0	Reference		Reference	
1	3.23 (1.14, 9.10)	0.027	2.49 (0.86, 7.25)	0.094
2	8.14 (2.50, 26.55)	0.001	4.85 (1.41, 16.70)	0.012
3	4.6 (0.71, 29.84)	0.11	1.80 (0.22, 14.79)	0.583
4	Empty		Empty	
Unknown	6.07 (2.03, 18.20)	0.001	4.52 (1.44, 14.13)	0.010
Previous ICI therapy	0.89 (0.3, 2.63)	0.827		
Surgery				
No Surgery	Reference		Reference	
Primary Surgery	0.41 (0.18, 0.94)	0.035	0.42 (0.17, 1.04)	0.060
Secondary Surgery	0.90 (0.40, 2.06)	0.812	1.02 (0.43, 2.43)	0.966
Immunosuppressive therapy for irAE	1.87 (1.0–3.51)	0.057		
History of Chronic Infections	2.79 (1.09, 7.11)	0.032	1.74 (0.61, 4.97)	0.304
Antibiotics/antivirals	3.62 (1.53, 8.53)	0.003	3.17 (1.16, 8.66)	0.024
GCSF use	0.88 (0.4, 1.95)	0.75		
Line of therapy				
1L	Reference			
2L	0.74 (0.40, 1.39)	0.349		
>2L	0.68 (0.22, 2.17)	0.519		
Therapy				
Monotherapy	Reference			
Combination	0.72 (0.42, 1.22)	0.223		
Number of cycles				
1	Reference		Reference	
2	0.5 (0.16–1.55)	0.23	0.44 (0.12, 1.54)	
3 or more	0.41 (0.18–0.89)	0.025	0.42 (0.17, 1.02)	

**Table 4:** Outcomes during pembrolizumab therapy. ED: Emergency Department.

	Infection			
	Yes	No	Total	*p*-Value
	*N* = 111	*N* = 132	*N* = 243	
Immune Related Adverse Events	33 (29.73%)	33 (25.00%)	66 (27.16%)	0.41
Colitis	4 (3.60%)	7 (5.30%)	11 (4.53%)	0.53
Adrenalitis	0 (0.00%)	1 (0.76%)	1 (0.41%)	0.36
Thyroiditis	8 (7.21%)	9 (6.82%)	17 (7.00%)	0.91
Pneumonitis	17 (15.32%)	7 (5.30%)	24 (9.88%)	0.009
Dermatitis	9 (8.11%)	13 (9.85%)	22 (9.05%)	0.64
Hepatitis	3 (2.70%)	0 (0.00%)	3 (1.23%)	0.057
Other	8 (7.21%)	5 (3.79%)	13 (5.35%)	0.24
ED Visit	37 (33.33%)	26 (19.70%)	63 (25.93%)	0.016
Inpatient	87 (78.38%)	53 (40.15%)	140 (57.61%)	<0.001
IMU/ICU admissions	26 (23.42%)	5 (3.79%)	31 (12.76%)	<0.001
Death	74 (66.67%)	70 (53.03%)	144 (59.26%)	0.031
Cause of Death				0.041
Primary Disease	49 (66.22%)	61 (87.14%)	110 (76.39%)	
Infection	11 (14.86%)	4 (5.71%)	15 (10.42%)	
Multiorgan Failure	9 (12.16%)	3 (4.29%)	12 (8.33%)	
Second Cancer	1 (1.35%)	0 (0.00%)	1 (0.69%)	
Other	4 (5.41%)	2 (2.86%)	6 (4.17%)	

## Data Availability

Data was made available through the charting review system Epic.

## Supplementary Materials

The following supporting information can be downloaded at: https://www.mdpi.com/article/10.3390/cancers15010081/s1, Figure S1: Distribution of infections; Figure S2A: Median overall survival based on monotherapy and chemotherapy in infected and non-infected patients; Figure S2B: Median overall survival based on infectious disease consultation status in infected patients; Table S1: Cultured infectious organisms based on infection site; Table S2: Outcomes of infection, anti-infectives used in treatment.

## Abbreviations

NSCLC	Non-small cell lung cancer
ICI	Immune checkpoint inhibitor
irAE	Immune related adverse event
ED	Emergency Department
IMU	Intermediate Care Unit
ICU	Intensive Care Unit
OS	Overall survival
PFS	Progression free survival
OR	Odds ratio
CI	Confidence interval
ECOG	Eastern Cooperative Oncology Group
PCR	Polymerase Chain Reaction
SSTI	soft tissue/skin infection
UTI	Urinary Tract Infection
HBV	Hepatitis B Virus
HCV	Hepatitis C Virus
HIV	Human Immunodeficiency
HNSCC	Head and Neck Squamous Cell Carcinoma
ESCMID	European Society of Clinical Microbiology and Infectious Diseases
RR	Relative Risk
PD-1	Programmed Death-1
