# Supplementary material for: Impact of Infections in Patients Receiving Pembrolizumab-Based Therapies for Non-Small Cell Lung Cancer
Source: Cancers (Basel). 2022 Dec 23;15(1):81. doi: 10.3390/cancers15010081 (PMC9817839; doi:10.3390/cancers15010081)
Supplement: Supplementary file 1 [file cancers-15-00081-s001.zip › cancers-2109633-supplementary.pdf]

cancers

MDPI

Supplementary Materials

Impact of Infections in Patients Receiving Pembrolizumab-Based Therapies for Non-Small Cell Lung Cancer

Ethan A. Burns, Kelly Gee, Ryan B. Kieser, Jiaqiong Xu, Yuqi Zhang, Aubrey Crenshaw, Ibrahim N. Muhsen, Charisma Mylavarapu, Abdullah Esmail, Shivan Shah, Godsfavour Umoru, Kai Sun, Carlo Guerrero, Zimu Gong, Kirk Heyne, Monisha Singh, Jun Zhang, Eric H. Bernicker and Maen Abdelrahim

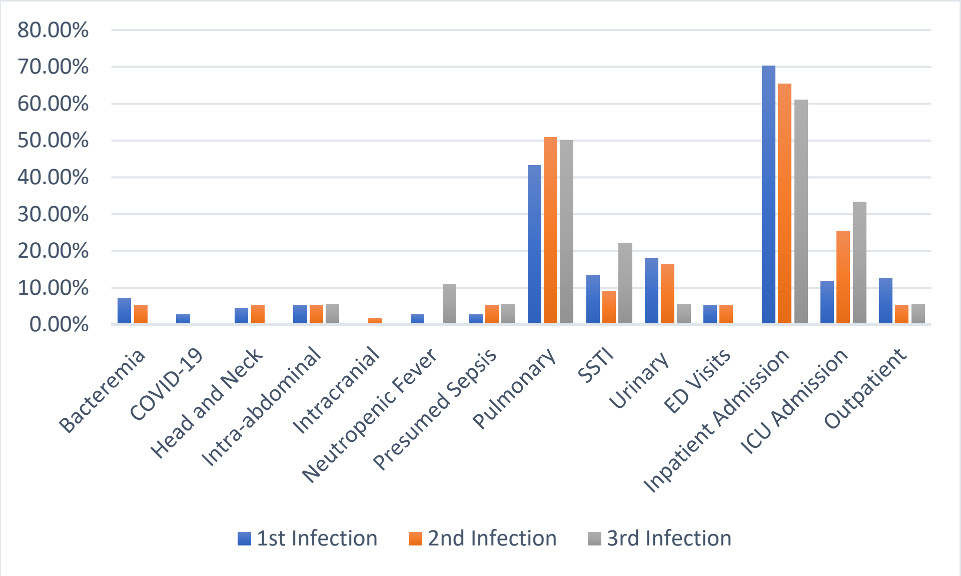

Figure S1. Distribution of each infection. SSTI: Skin and soft tissue infection; ED: Emergency Department; ICU: Intensive Care Unit.

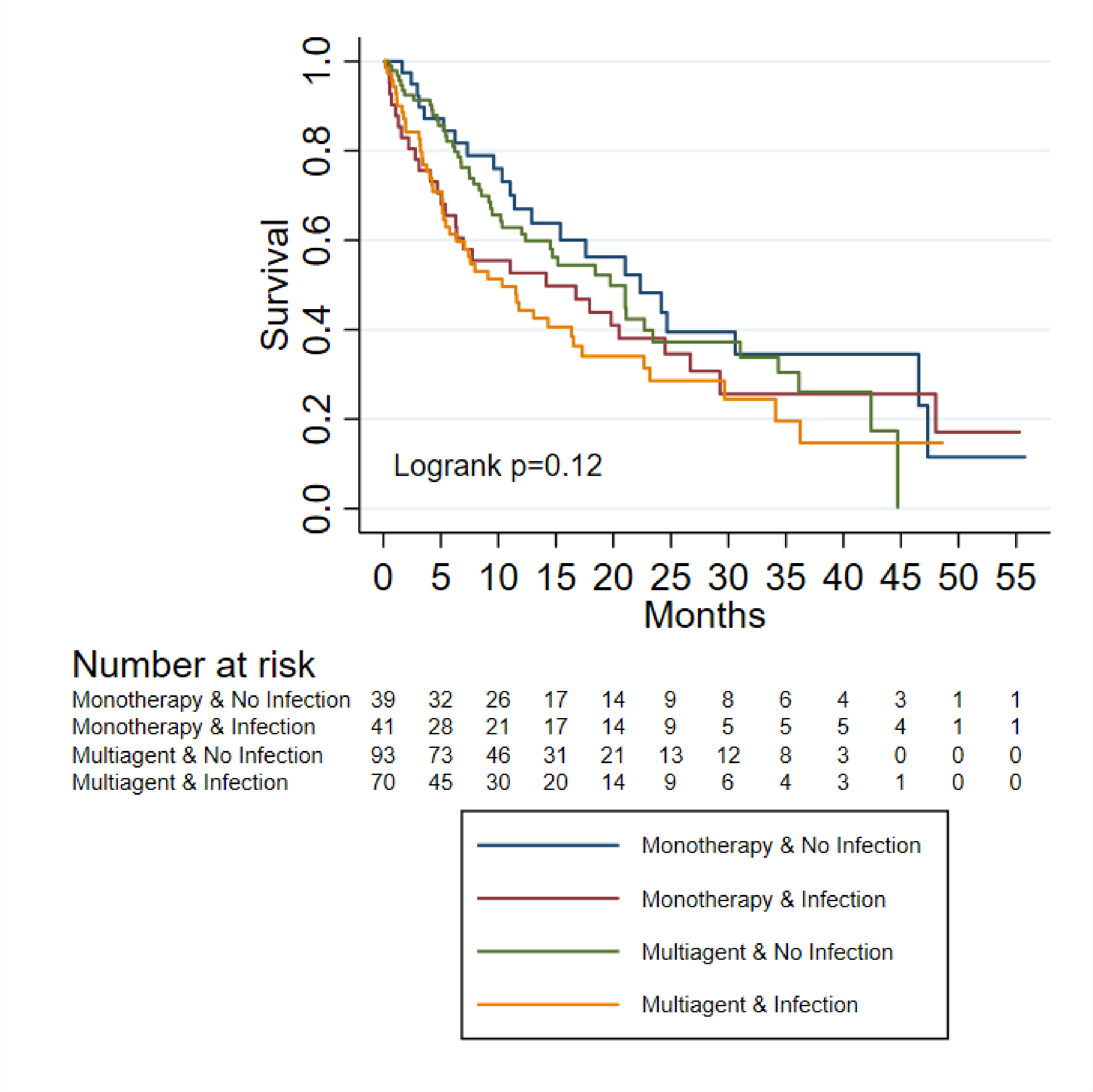

Figure S2A. Median overall survival for monotherapy and chemotherapy for infected and noninfected patients.

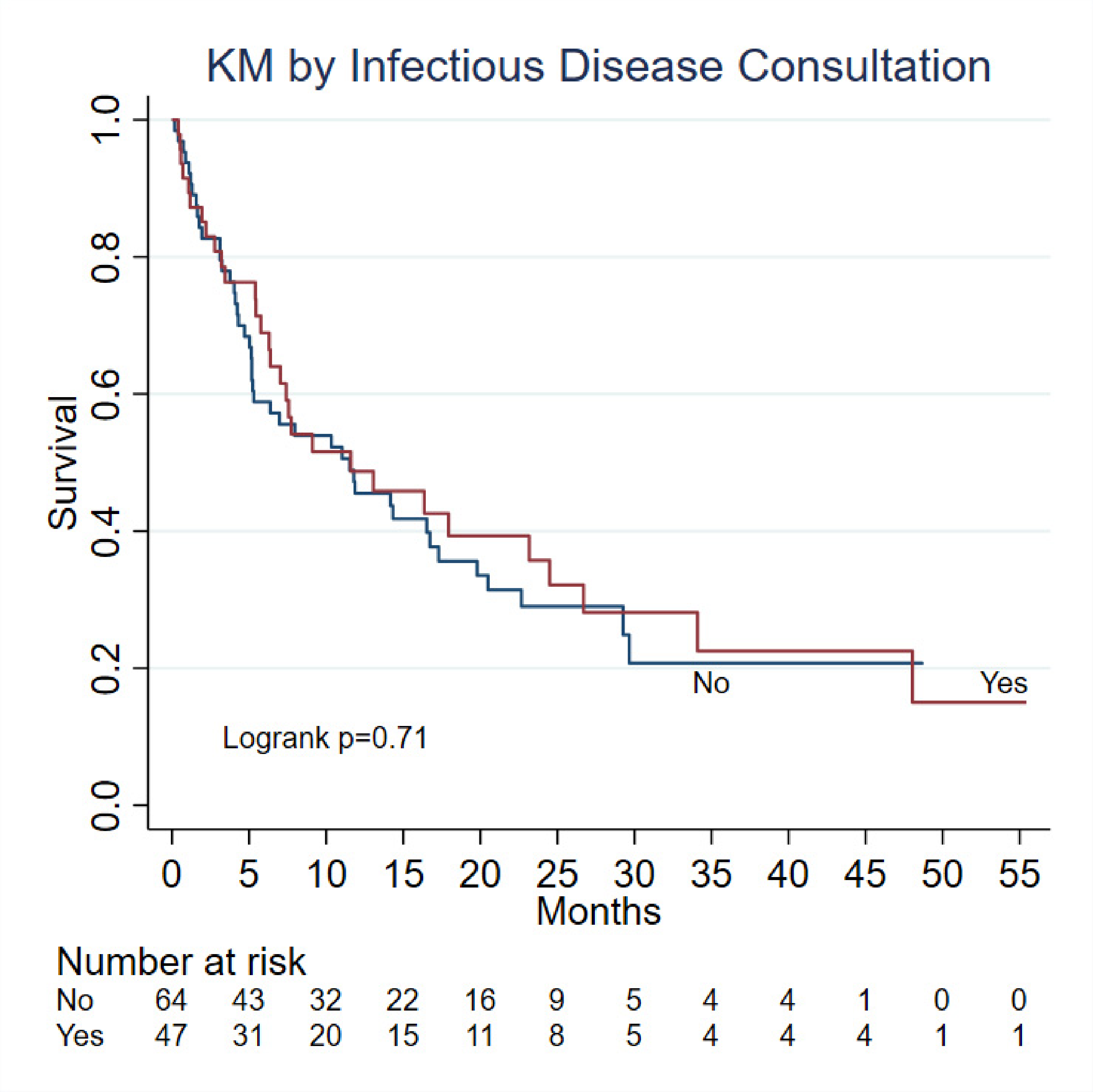

Figure S2B. Survival for patients who received infectious disease consultation.

Table S1. Distribution of culture positive infections.

Type of infection	Pathogen	1st Infection	2nd Infection	3rd Infection
Bacteremia/Fungemia				
Total Number of Cases		8	3	1
	Pathogen Isolated	8	3	1
	Corynebacterium species	1	0	0
	Escherichia coli	1	0	0
	Micrococcus luteus	1	0	0
	Proteus mirabilis	1	0	0
	Staphylococcus Aureus	1	1	0
	Staphylococcus epidermidis	1	0	0
	Staphylococcus hominis	1	0	0
	Bacillis lentis	0	1	0

	Streptococcus pneumoniae	1	0	1
	Candida Tropicalis	0	1	0
Pulmonary				
Total Number of Cases		49	29	9
	Pathogen Isolated	13	8	3
	Coronavirus HKU1	2	0	0
	COVID 19	3	0	0
	Influenza B	1	1	1
	RSV	1	0	0
	Rhinovirus/Enterovirus	4	1	1
	Parainfluenza-1	0	1	0
	Escherichia coli	1	0	0
	Escherichia coli, Legionella	0	1	0
	Sternotrophomonas	0	1	0
	Staphylococcus aureus	0	0	1
	Pseudomonas aeruginosa	1	2	0
	Aspergillosis	0	1	0
Neutropenic Fever				
Total Number of Cases		3	0	2
	Pathogen Isolated	0	0	0
Head and Neck				
Total Number of Cases		5	5	0
	Pathogen Isolated	2	2	0
	Candida Esophagitis	2	2	0
Intraabdominal				
Total Number of Cases		6	3	1
	Pathogen Isolated	6	1	0
	Clostridium difficil	5	0	1
	Enteropathogenic Escherichia coli	1	1	0
Soft Tissue Skin Infection				
Total Number of Cases		15	5	4
	Pathogen Isolated	6	2	0
	Enterobacter cloacae	0	1	0
	Enterococcus faecalis, Staphylococcus aureus, Prevotella melaninogenica	1	0	0
	Herpes simplex-1	3	0	0
	Staphylococcus aureus, Proteus mirabilis	1	0	0
	Varicella zoster reactivation	1	1	0
Urinary Tract Infection				
Total Number of Cases		20	10	1
	Pathogen Isolated	18	6	1
	Candida Albicans	1	0	0
	Citrobacter koseri	1	0	0
	Escherichia coli	6	4	1
	Escherichia coli, Klebsiella pneumoniae	1	0	0
	Escherichia coli, Enterococcus faecalis	1	0	0
	Enterobacter cloacae	1	0	0

	Enterococcus faecalis	1	1	0
	Enterococcus faecium	1	0	0
	Klebsiella pneumoniae	1	0	0
	Proteus mirabilis	1	1	0
	Pseudomonas aeruginosa	2	0	0
	Streptococcus sanguinis	1	0	0
Presumed Sepsis				
Total Number of Cases		3	2	1
	Pathogen Isolated	0	0	0
Intracranial				
	Pathogen Isolated	0	1	0
	Serratia marscenses	0	1	0

Table S2. Outcome of Infection.

Outcome	Total (n=111)
Treatment Delay	56 (50.4%)
Treatment Discontinuation	28 (25.2%)
Progression Documented During Infection	16 (14.4%)
Infection-Related Death	11 (9.9%)
Anti-Infectives for Infections	
Antimicrobials	104 (93.7%)
Antiviral	12 (10.8%)
Antifungal	10 (9.0%)
Antimicrobials	
Total	216
Azithromycin	9 (4.2%)
Penicillins	29 (13.4%)
Cephalosporin	64 (29.6%)
Vancomycin	53 (24.5%)
Metronidazole	9 (4.2%)
Fluoruoquinolone	23 (10.6%)
Aztreonam	2 (0.9%)
Tetracycline	8 (3.7%)
Linezolid	2 (0.9%)
Carbopenem	11 (5.1%)
Clindamycin	2 (0.9%)
Nitrofurantoin	1 (0.5%)
Tobramycin	1 (0.5%)
Daptomycin	1 (0.5%)
Trimethoprim-Sulfamethaxazole	1 (0.5%)
Antiviral	
Total	10
Oseltamivir	3 (30.0%)
Acyclovir	2 (20.0%)
Valacyclovir	2 (20.0%)
Remdesivir	3 (30.0%)
Antifungal	
Total	10
Fluconazole	8 (80.0%)
Voriconazole	1 (10.0%)
Micafungin	1 (10.0%)
